# The independent and combined associations between the intake of ultra-processed foods, sedentary behavior, and depressive symptoms in young adults

**DOI:** 10.3389/fnut.2025.1675892

**Published:** 2025-10-13

**Authors:** Jiajia Ren, Lei Zhou, Yize Li, Shuo Zhang, Xiaoyu Xu, Xinli Chi, Hong Xie

**Affiliations:** ^1^School of Public Health, Bengbu Medical University, Bengbu, China; ^2^School of Psychology, Shenzhen University, Shenzhen, China

**Keywords:** ultra-processed food, sedentary behavior, depressive symptoms, combined effects, young population

## Abstract

**Background:**

Ultra-processed food (UPF) intake and sedentary behavior (SB) are recognized as significant contributors to depressive symptoms. However, the joint effects of these two factors on depressive symptoms remain insufficiently explored. This study aimed to investigate the independent and combined associations of UPF intake and SB with depressive symptoms in a young population.

**Methods:**

Cross-sectional analyses were conducted using baseline survey data from the Shenzhen Youth Health Cohort. UPF intake, SB, and depressive symptoms were self-reported via questionnaires. Multifactorial logistic regression models were employed to assess the independent and joint associations between UPF intake, SB, and depressive symptoms.

**Results:**

A total of 1,461 participants were included, with 610 (41.8%) reporting depressive symptoms and 851 (58.2%) without. Compared with the group with the lowest UPF supply ratio (Q1), the risk of depressive symptoms was significantly higher in the third (Q3) and fourth (Q4) quartiles, with odds ratios (95% CI) of 1.60 (1.16–2.21) and 2.05 (1.48–2.85), respectively. Similarly, individuals with SB exceeding 8 h per day had a significantly increased risk of depressive symptoms compared to those with SB of less than 4 h per day (OR = 1.75, 95% CI: 1.25–2.44). In the combined analysis, participants with high UPF intake and sedentary time ≥6 h per day exhibited the highest risk of depressive symptoms compared to those with low UPF intake and sedentary time <6 h per day (OR = 2.31, 95% CI: 1.62–3.31).

**Conclusion:**

Higher intake of UPF and increased SB were significantly associated with depressive symptoms in the young population. Additionally, the combination of high UPF intake and sedentary time exceeding 6 h per day was associated with a further heightened risk of depressive symptoms.

## 1 Introduction

Depressive symptoms represent early or mild forms of depression, where the severity and duration typically do not meet the diagnostic criteria for clinical depression. Individuals may experience transient sadness, loss of interest, feelings of hopelessness, and anxiety (1). While depressive symptoms may gradually resolve over time or with changes in environmental circumstances, persistent symptoms pose a risk not only for the development of depression but also for other issues (2), such as suicidal tendencies (3). According to a report released by the Institute of Psychology at the Chinese Academy of Sciences in 2023 (4), the prevalence of depressive symptoms in the adult population was 10.6%, with the highest rates observed in the youth demographic, particularly among those aged 18–24 and 25–34 years. Within these age groups, the detection rates were notably high at 24.1 and 12.3%, respectively, significantly surpassing those in other age brackets.

Concurrent with socioeconomic development, shifts in lifestyle behaviors—particularly diet and SB—have emerged as novel contributors to the onset of depressive symptoms. As diet plays a pivotal role in mental health (5, 6), the relationship between ultra-processed food (UPF) intake and depressive symptoms has garnered growing scientific and public attention. Multiple studies have demonstrated that high UPF consumption is associated with an elevated risk of depressive symptoms compared to lower UPF intake (7–9). However, research examining the association between UPF intake and depressive symptoms specifically within youth populations remains limited. On the other hand, sedentary behavior (SB) is an established health risk factor independent of physical activity (10). Previous research has indicated that SB is positively correlated with an increased risk of depressive symptoms (11). Furthermore, studies have shown that sedentary time exceeding 6 h per day significantly increases the risk of depressive symptoms when compared to sedentary time of less than 2 h per day (12).

Health risk behaviors are often synergistic, with combinations of two or more behaviors frequently linked to a higher risk of chronic diseases than each behavior individually (13). Evidence suggests that UPF intake and SB may mutually reinforce one another, with individuals who spend more time sedentary more likely to consume higher amounts of UPF (14), and UPF intake potentially promoting SB (15). However, most existing research has focused on the effects of individual factors on depressive symptoms, leaving the combined influence of UPF intake and SB largely unexplored.

Thus, utilizing data from the Shenzhen youth population, this study aims to investigate the independent and joint associations of UPF intake, SB, and depressive symptoms within this demographic.

## 2 Methods

### 2.1 Sample source

This study is based on a baseline survey conducted within the Shenzhen Youth Population Health Cohort. The primary aim of the cohort is to identify potential risk and protective factors for disease, including social characteristics, behavioral performance, lifestyle choices, health literacy, and psychological traits. Additionally, the cohort seeks to describe the pathways of health changes and their underlying mechanisms from a multidimensional perspective, while providing a scientific foundation for the development of targeted health strategies that account for the unique health ecology of Shenzhen. Participants were recruited between December 2023 and October 2024 from the physical examination centers of two tertiary hospitals in Longhua District, Shenzhen. A cross-sectional survey was conducted through the use of QR codes and electronic questionnaires. The study received approval from the Medical Committee of the Science and Technology Ethics Committee of Tsinghua University (Ethics No. 20230065) prior to commencement.

### 2.2 Inclusion and exclusion criteria

(1) Inclusion Criteria: Participants aged 18–35 years who provided signed informed consent.

(2) Exclusion Criteria: Individuals with a history of major metabolic or serious illnesses (e.g., cancer, stroke); individuals with cognitive impairments preventing cooperation; individuals with missing dietary data; and those with extreme total energy intake, defined as energy intake below the 2.5th percentile or above the 97.5th percentile of the sex-specific distribution in our sample, a method commonly used in nutritional epidemiological studies (16). The final cohort comprised 1,461 participants.

### 2.3 UPF intake assessment

Dietary intake was assessed using a self-administered Food Frequency Questionnaire (FFQ), developed by the research team. The questionnaire was based on the 6th edition of the Chinese Food Composition Table (Standard Edition) and the NOVA Food Classification System (17). The FFQ was adapted from the instrument used in the China Health and Nutrition Examination Survey cohort study (18). It includes 10 food categories: staple foods, legumes, vegetables, fruits, meat, eggs, dairy, alcoholic beverages and drinks, snacks, and condiments. The Cronbach’s *α* coefficient for the FFQ in this study was 0.974.

UPF intake was evaluated using the UPF energy supply ratio (19), which is calculated as:


$$\mathrm{UPF}\;\text{energy supply ratio}=\frac{\mathrm{UPF}\;\text{energy intake}}{\text{total energy intake}}\times 100\%$$

Participants were then categorized into quartiles based on the UPF energy supply ratio: Q1 (UPF energy supply ratio <23.2%), Q2 (23.2–38.8%), Q3 (38.8–53.3%), and Q4 (>53.3%).

### 2.4 Assessment of SB

SB over the previous 7 days was assessed using the International Physical Activity Questionnaire (IPAQ) short form (20). Based on prior studies, SB was classified into four categories: <4 h/day, 4–6 h/day, 6–8 h/day, and >8 h/day (21).

### 2.5 Assessment of depressive symptoms

Depressive symptoms were assessed using the Patient Health Questionnaire-9 (PHQ-9) for the previous 2 weeks. The PHQ-9 comprises 9 items, each scored on a 4-point Likert scale, with a total score range of 0–27 (22). Higher scores indicate greater severity of depressive symptoms. A score of 0–4 indicates no depressive symptoms, 5–9 suggests mild depressive symptoms, 10–14 indicates moderate depressive symptoms, 15–19 reflects moderately severe depressive symptoms, and 20–27 suggests severe depressive symptoms. For this study, a PHQ-9 score of ≥5 was considered indicative of depressive symptoms (23).

### 2.6 Covariates

The general questionnaire collected information on several covariates, including demographic characteristics (age, gender, education level, income status), mental health status (history of mental illness, medication use for mental health conditions), lifestyle behaviors (smoking, alcohol consumption, sleep duration, physical activity), and body mass index (BMI, kg/m^2^). Educational attainment was categorized into three levels: high school and below, college or bachelor’s degree, and master’s degree or above. Income status was classified as insufficient, balanced, or sufficient. Physical activity levels were grouped into low, medium, and high categories.

### 2.7 Statistical analysis

Normality tests indicated that all continuous variables were non-normally distributed. Non-normally distributed continuous variables were presented as medians (interquartile range), with differences between groups assessed using the Wilcoxon rank sum test and Kruskal-Wallis test. Categorical variables were reported as frequencies (proportions), and differences between groups were evaluated using the χ^2^-test. Multifactorial logistic regression models were applied to assess the associations between UPF intake, sedentary behavior, and depressive symptoms. The models were as follows: Model 1 (unadjusted); Model 2 (adjusted for age, gender, education level, income status, BMI, history of mental illness, and history of medication use for mental health conditions); and Model 3 (further adjusted for smoking, alcohol consumption, physical activity, sleep duration, and energy intake, based on Model 2). A three-node (10th, 50th, and 90th percentiles) Restricted Cubic Splines (RCS) model was employed to evaluate the dose–response relationship between UPF intake, SB, and depressive symptoms.

Joint analyses were conducted by combining UPF intake (low: UPF supply ratio ≤ 38.8%, high: UPF supply ratio >38.8%) and SB (sedentary time <6 h/day; sedentary time ≥ 6 h/day), with the reference group consisting of those with low UPF intake and sedentary time <6 h/day. The interaction between UPF intake and SB in relation to depressive symptoms was further examined. Finally, subgroup and sensitivity analyses were performed to assess the robustness of the findings. Statistical analyses were conducted using SPSS (version 23.0) and R (version 4.3.2), with a significance level set at *α* = 0.05 and two-sided tests.

## 3 Results

### 3.1 Participant characteristics

As shown in Table 1, a total of 1,461 participants were included in this study, of which 610 (41.8%) reported depressive symptoms, and 851 (58.2%) did not. Energy intake was significantly higher in participants with depressive symptoms compared to those without (*p* < 0.001). Compared with individuals without depressive symptoms, those with depressive symptoms were more likely to have a college or bachelor’s degree, insufficient income, a history of mental illness and psychiatric medication use, smoking habits, alcohol consumption, sleep duration of less than 6 h, SB exceeding 8 h per day, and higher proportions in the Q3 and Q4 UPF energy supply ratio groups. No statistically significant differences were observed between depressive symptoms and factors such as age, gender, marital status, BMI, and physical activity levels (*p* > 0.05).

**Table 1:** Descriptive characteristics of participants by depressive symptoms status.

Variables	*N* = 1,461	Depressive symptoms		*P*
		Yes (*n* = 610)	No (*n* = 851)	
Age (years)	24.0 (22.0, 26.0)	24.0 (22.0, 26.0)	24.0 (22.0, 26.0)	0.805
Gender				0.127
Male	558	219(35.9)	339(39.8)	
Female	903	391(64.1)	512(60.2)	
Education level				0.020
High school and below	151	69(11.3)	82(9.6)	
College or bachelor’s degree	1,185	503(82.5)	682(80.1)	
Master’s degree or above	125	38(6.2)	87(10.2)	
Marital status				0.101
Married	622	275(45.1)	347(40.8)	
Others	839	335(54.9)	504(59.2)	
Income status				<0.001
Insufficient	396	201(33.0)	195(22.9)	
Balanced	531	204(33.4)	327(38.4)	
Sufficient	534	205(33.6)	329(38.7)	
BMI (kg/m^2^)	21.2 (19.4, 24.1)	21.2 (19.4, 24.1)	21.3 (19.4, 24.1)	0.914
History of mental illness				<0.001
No	1,349	526(86.2)	823(96.7)	
Yes	112	84(13.8)	28(3.3)	
Medication use for mental health conditions				<0.001
No	1,402	566(92.8)	836(98.2)	
Yes	59	44(7.2)	15(1.8)	
Smoking				<0.001
No	1,103	425(69.7)	678(79.7)	
Yes	358	185(30.3)	173(20.3)	
Alcohol consumption				0.015
No	1,133	454(74.4)	679(79.8)	
Yes	328	156(25.6)	172(20.2)	
Sleep duration				<0.001
<6 h	96	63(10.3)	33(3.9)	
6-8 h	1,248	500(82.0)	748(87.9)	
>8 h	117	47(7.7)	70(8.2)	
Physical activity				0.187
Low	355	159(26.0)	196(23.0)	
Medium	900	375(61.5)	525(61.7)	
High	206	76(12.5)	130(15.3)	
SB (h/d)				<0.001
<4	301	118(19.3)	183(21.5)	
4–6	232	84(13.8)	148(17.4)	
6–8	543	212(34.8)	331(38.9)	
>8	385	196(32.1)	189(22.2)	
UPF intake				<0.001
Q1	365	110(18.0)	255(30.0)	
Q2	365	138(22.6)	227(26.7)	
Q3	365	168(27.5)	197(23.1)	
Q4	366	194(31.8)	172(20.2)	
Total energy intake (kcal/d)	2461.9(1553.1, 4086.3)	2656.5(1614.6, 4957.1)	2356.1(1491.1, 3675.8)	<0.001

Continuous variables are presented as medians (interquartile range). Categorical variables are presented as numbers (percentages). BMI, body mass index; SB, sedentary behavior; UPF, ultra-processed food.

### 3.2 Association of UPF intake and SB with depressive symptoms

**Figure 1:**
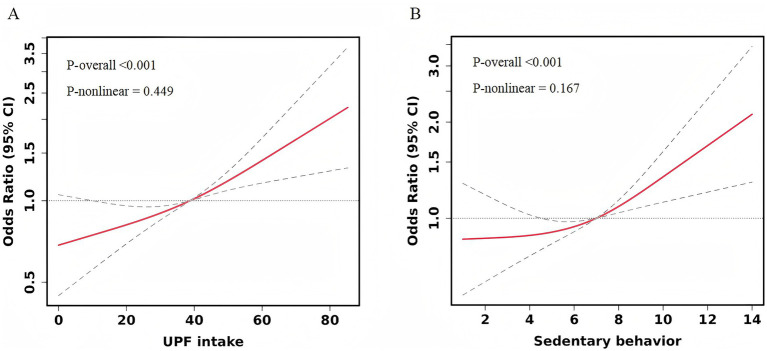
Restricted cubic spline curve for the association between UPF intake, SB, and depressive symptoms. Adjusted for age, gender, education level, income status, BMI, history of mental illness, medication use for psychiatric conditions, smoking, alcohol consumption, physical activity, sleep duration, and total energy intake. **(A)** UPF intake. **(B)** Sedentary behavior.

Logistic regression models were employed to analyze the associations between UPF intake, SB, and depressive symptoms. In the unadjusted model (Table 2), the risk of depressive symptoms increased across the higher quartiles of UPF intake when compared to the lowest quartile (Q1). The odds ratios (ORs) with 95% confidence intervals (CIs) for Q2, Q3, and Q4 were 1.41 (1.04–1.92), 1.98 (1.46–2.68), and 2.61 (1.93–3.55), respectively. After adjusting for confounding factors, including age, gender, education level, income status, BMI, history of mental illness, medication use for psychiatric conditions, smoking, alcohol consumption, physical activity, sleep duration, and total energy intake, the risk remained significantly higher in the Q3 and Q4 groups compared to the Q1 group, with ORs of 1.60 (1.16–2.21) and 2.05 (1.48–2.85), respectively. No significant association was observed between the Q2 group and depressive symptoms. Restricted Cubic Splines (RCS) analysis (Figure 1A) revealed no nonlinear dose–response relationship between UPF intake and depressive symptom risk (P for nonlinear = 0.449).

**Table 2:** Association analysis of UPF intake, SB, and depressive symptoms.

Variables	Model 1		Model 2		Model 3	
	*OR*(95%*CI*)	*P*	*OR*(95%*CI*)	*P*	*OR*(95%*CI*)	*P*
UPF intake						
Q1	1.00		1.00		1.00	
Q2	1.41(1.04–1.92)	0.029	1.35(0.99–1.86)	0.060	1.27(0.92–1.76)	0.139
Q3	1.98(1.46–2.68)	<0.001	1.79(1.31–2.46)	<0.001	1.60(1.16–2.21)	0.004
Q4	2.61(1.93–3.55)	<0.001	2.35(1.72–3.23)	<0.001	2.05(1.48–2.85)	<0.001
SB						
<4 h/d	1.00		1.00		1.00	
4–6 h/d	0.88(0.62–1.25)	0.480	0.97(0.68–1.40)	0.885	0.95(0.65–1.38)	0.783
6–8 h/d	0.99(0.74–1.33)	0.964	1.07(0.79–1.45)	0.667	1.08(0.79–1.48)	0.618
>8 h/d	1.61(1.19–2.19)	0.002	1.79(1.30–2.48)	<0.001	1.75(1.25–2.44)	0.001

Logistic regression models were used to calculate odds ratios (ORs) and 95% confidence intervals (CIs). Model 1: unadjusted. Model 2: adjusted for demographic characteristics (age, gender, education level, income status); mental health status (history of mental illness, medication use for mental health conditions); and BMI. Model 3: further adjusted for lifestyle behaviors (smoking, alcohol consumption, sleep duration, physical activity) and total energy intake. UPF, ultra-processed food; SB, sedentary behavior.

In relation to SB, when not adjusted for confounders, the most sedentary group (sedentary time >8 h/day) had a significantly higher risk of depressive symptoms compared to the least sedentary group (sedentary time <4 h/day), with an OR of 1.61 (95% CI: 1.19–2.19). After adjusting for all covariates, the risk of depressive symptoms remained significantly higher in the highest sedentary time group compared to the lowest sedentary time group, with an OR of 1.75 (95% CI: 1.25–2.44). Sedentary time categories of 4–6 h/day and 6–8 h/day did not show significant associations with depressive symptoms in any of the models, as detailed in Table 2. Additionally, no nonlinear dose–response relationship was observed between SB and depressive symptom risk (P for nonlinear = 0.167) (Figure 1B).

### 3.3 Combined effects of UPF intake and SB on depressive symptoms

The combined effects of UPF intake and SB were analyzed across four subgroups (Table 3): Group A (low UPF intake and sedentary time <6 h/day, *n* = 242), Group B (low UPF intake and sedentary time ≥6 h/day, *n* = 488), Group C (high UPF intake and sedentary time <6 h/day, *n* = 291), and Group D (high UPF intake and sedentary time ≥6 h/day, *n* = 440). In Model 3, participants with high UPF intake and sedentary time ≥6 h/day exhibited the greatest risk of depressive symptoms compared to those with low UPF intake and sedentary time <6 h/day, with an OR of 2.31 (95% CI: 1.62–3.31). The risk of depressive symptoms was also significantly increased in the other subgroups, with ORs of 1.46 (95% CI: 1.03–2.08) and 1.72 (95% CI: 1.17–2.52) for Groups B and C, respectively. Additionally, the interaction between UPF intake and SB on depressive symptoms was not statistically significant (Supplementary Table 1).

**Table 3:** Analysis of the combined effect of UPF intake and SB on depressive symptoms.

Group	n(%)	Model 1		Model 2		Model 3	
		*OR*(95%*CI*)	*P*	*OR*(95%*CI*)	*P*	*OR*(95%*CI*)	*P*
A	242(16.6)	1.00		1.00		1.00	
B	488(33.4)	1.50(1.07–2.10)	0.019	1.51(1.07–2.14)	0.020	1.46(1.03–2.08)	0.035
C	291(19.9)	2.18(1.52–3.15)	<0.001	1.96(1.35–2.85)	<0.001	1.72(1.17–2.52)	0.006
D	440(30.1)	2.75(1.97–3.87)	<0.001	2.58(1.82–3.67)	<0.001	2.31(1.62–3.31)	<0.001

Logistic regression models were used to calculate odds ratios (ORs) and 95% confidence intervals (CIs). Group A: Low UPF intake and sedentary time <6 h/day (Reference group). Group B: Low UPF intake and sedentary time ≥6 h/day. Group C: High UPF intake and sedentary time <6 h/day.·Group D: High UPF intake and sedentary time ≥6 h/day. Model adjustments: Model 1: Unadjusted. Model 2: Adjusted for demographic characteristics (age, gender, education level, income status), mental health status (history of mental illness, medication use for mental health conditions), and BMI. Model 3: Additionally adjusted for lifestyle behaviors (smoking, alcohol consumption, sleep duration, physical activity) and total energy intake. UPF, ultra-processed food; SB, sedentary behavior.

### 3.4 Subgroup and sensitivity analysis

**Figure 2:**
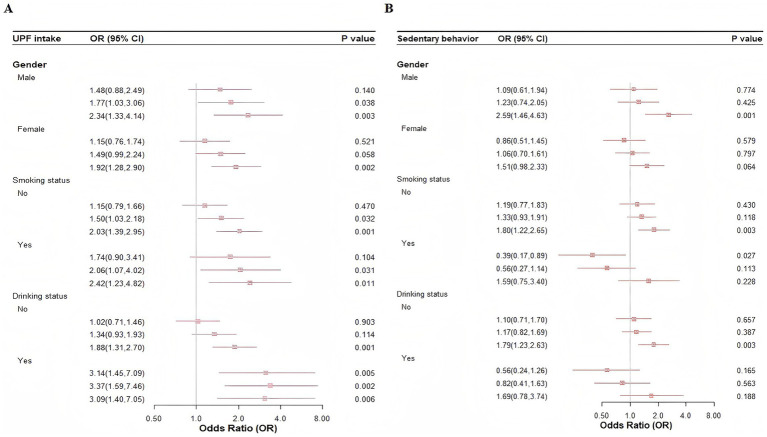
Subgroup analysis of the association between UPF intake, SB, and depressive symptoms. **(A)** UPF intake. **(B)** Sedentary behavior.

Subgroup analyses based on gender (male/female), smoking status (yes/no), and alcohol consumption (yes/no) revealed similar associations between UPF intake and depressive symptoms (Figure 2A). However, no significant associations were observed between SB and depressive symptoms among non-smokers and non-drinkers (Figure 2B). Sensitivity analyses further confirmed the consistency of the results when age (early youth/late youth) and BMI (underweight/normal weight/overweight or obese) were categorized (Supplementary Tables 2, 3).

## 4 Discussion

Our findings suggest that, in young populations, both higher intake of UPF and increased SB are significantly associated with an elevated risk of depressive symptoms. Furthermore, the combination of high UPF intake and sedentary time exceeding 6 h per day further exacerbates this risk, as demonstrated in the joint analysis.

These results align with several previous epidemiological studies. A cohort study of 26,730 adults revealed that higher UPF intake was positively associated with an increased risk of depressive symptoms (24). Two subsequent cross-sectional studies found that individuals in the highest quartile of UPF consumption had higher odds of developing depressive symptoms compared to those in the lowest quartile (8, 9). However, these studies were primarily conducted in countries such as France (24), the United States (9), Italy (8), Spain (25), and Brazil (26), and mostly involved adult populations. Our study, focusing on young adults in Shenzhen, China, supports these findings and extends the knowledge in this area by demonstrating that higher UPF intake is also associated with an increased risk of depressive symptoms among young adults.

The potential mechanisms underlying the association between UPF intake and depressive symptoms are multifaceted. Firstly, UPF is characterized by high levels of sugar, salt, saturated fat, and energy, while being low in protein, dietary fiber, vitamins, and minerals, resulting in poor nutritional quality (27). Diets with these components can induce dysbiosis, disrupt gut barrier function, and alter neurotransmitter metabolism both in the gut and brain, ultimately affecting brain function and behavior (28). Secondly, various food additives in UPF may contribute to depressive symptoms (29). For example, artificial sweeteners (e.g., aspartame, saccharin) may alter the synthesis and release of neurotransmitters such as dopamine, norepinephrine, and serotonin, which are critical to mental health (30). Additionally, bisphenol A (commonly used in the production of plastic food and beverage containers) disrupts stress-sensitive and endocrine systems, potentially contributing to depressive states later in life (31).

Our study also found that individuals in the highest sedentary time group (>8 h/day) had a significantly increased risk of depressive symptoms compared to those in the lowest sedentary time group (<4 h/day). A previous study involving adolescents aged 12–15 years (*N* = 67,077) across 30 low- and middle-income countries found that the prevalence of depressive symptoms increased linearly with sedentary time, with behaviors exceeding 1–2 h per day associated with a higher risk of depression, regardless of physical activity levels (32). A separate cross-sectional study conducted in China (*N* = 11,787) found that nearly 50% of college students with screen time exceeding 4 h per day reported depressive symptoms, with this association being significantly stronger for screen time >4 h/day compared to ≤2 h/day (33).

In our subgroup analyses, no significant associations were found between SB and depressive symptoms among non-smokers and non-drinkers. Several factors may explain this. First, the insufficient sample size in these subgroups could have limited the statistical power to detect a significant effect. Second, chronic smoking may induce depressive symptoms through neuroendocrine and dopaminergic pathways (34), potentially masking the effect of SB (35, 36). Similarly, a J-shaped relationship between alcohol consumption and depressive symptoms has been suggested in existing research, where light or moderate alcohol consumption may have protective effects, while heavy consumption exacerbates depressive symptoms (37–39). These dual influences may reduce the observed relationship between SB and depressive symptoms in these groups.

There are several potential mechanisms explaining the relationship between SB and depressive symptoms. The social withdrawal hypothesis suggests that excessive time spent on passive, non-social activities (e.g., internet use, watching TV, or listening to music) may reduce social engagement, leading to social withdrawal behaviors that are strongly associated with the development of depression (40, 41). Furthermore, SB may mediate depression through inflammatory pathways (42, 43). Research has shown that SB increases C-reactive protein levels, which are associated with the development of depressive symptoms (44).

Additionally, our study observed that the combination of high UPF intake and sedentary time >6 h/day significantly increased the risk of depressive symptoms. However, no significant interaction between UPF intake and SB was found in relation to depressive symptoms. Several studies have proposed mechanisms that could explain this. First, there may be a mutually reinforcing relationship between SB and UPF intake. A previous study have shown that individuals with sedentary time >2 h/day had a higher daily consumption of UPF (42.8%) compared to those with less SB (29.8%) (45). Moreover, increased UPF intake is associated with higher levels of sedentary activities, such as watching TV, playing video games on weekends, and using smartphones during weekdays (46). This mutual reinforcement may further heighten the risk of depressive symptoms. In addition, both UPF intake and SB are linked to inflammatory responses (43, 47), which are a key feature of depression (42), and these factors may work synergistically to increase the risk of depression via inflammatory mechanisms.

Notably, the potential for reverse causality must also be considered, as depressive symptoms may themselves lead to poorer lifestyle choices. Previous studies have shown that depressive symptoms—such as low mood, diminished interest, and lack of energy—can lead individuals to withdraw from physical activity, thereby increasing sedentary behavior (48–51). Concurrently, depressive symptoms are positively correlated with unhealthy dietary patterns, including increased consumption of saturated fats, sugars, and emotional eating behaviors (52). From a physiological perspective, depression activates the hypothalamic–pituitary–adrenal (HPA) axis, leading to increased glucocorticoid release (53), which in turn stimulates appetite—particularly for highly palatable, high-fat, and high-sugar foods such as ultra-processed foods (54, 55). Thus, the associations identified in this study may be partly attributable to the effects of depressive symptoms on behavior. Further longitudinal and intervention studies are needed to clarify the temporal and causal pathways linking UPF intake, sedentary behavior, and depressive symptoms, thereby providing a robust scientific basis for effective mental health promotion strategies among young adults.

## 5 Strengths and limitations

To the best of our knowledge, this is the first study to examine the association between UPF intake and depressive symptoms in a young population in Shenzhen, China. Furthermore, we explored the combined effects of UPF intake and SB on depressive symptoms. However, several limitations should be considered. First, the study relied on self-reported questionnaires to assess sedentary time, which may have introduced recall bias and potentially affected the accuracy of the findings. Future research should validate these results by incorporating both accelerometer measurements and self-report methods. Second, the study was conducted in Longhua District, Shenzhen, and the sample may not be sufficiently representative of the broader youth population, limiting the generalizability of the findings to other regions or populations. Third, as a cross-sectional study, our design precludes the inference of causality. Longitudinal studies are necessary to confirm the observed associations and establish causal relationships.

## 6 Conclusion

In conclusion, we found that both higher UPF intake and increased SB were significantly associated with depressive symptoms in the young population. The combination of high UPF intake and sedentary time exceeding 6 h per day was linked to an even greater risk of depressive symptoms. These findings highlight the need for mental health interventions targeting youth to address both UPF consumption and SB. Comprehensive and integrated approaches are essential to mitigate the risk of depressive symptoms in this population.

## Data Availability

The raw data supporting the conclusions of this article will be made available by the authors, without undue reservation. Requests to access these datasets should be directed to Hong Xie, xh@bbmu.edu.cn.
