# Supplementary material for: The independent and combined associations between the intake of ultra-processed foods, sedentary behavior, and depressive symptoms in young adults
Source: Front Nutr. 2025 Oct 13;12:1675892. doi: 10.3389/fnut.2025.1675892 (PMC12554551; doi:10.3389/fnut.2025.1675892)
Supplement: Supplementary file 1 [file Table_1.DOCX]

**Supplementary Table 1**. A multiplicative interaction analysis of UPF intake and SB on depressive symptoms.

| **UPF intake** | **SB** | ***OR*(95%*CI*)** | ***P*** |
| --- | --- | --- | --- |
| Q1 | <4h/d | Ref |  |
| Q2 | 4-6h/d | 1.38(0.42, 4.52) | 0.387 |
| Q2 | 6-8h/d | 1.23(0.48, 3.13) | 0.614 |
| Q2 | >8h/d | 0.89(0.33, 2.37) | 0.857 |
| Q3 | 4-6h/d | 1.71(0.57, 5.20) | 0.497 |
| Q3 | 6-8h/d | 1.33(0.55, 3.17) | 0.893 |
| Q3 | >8h/d | 0.83(0.32, 2.12) | 0.421 |
| Q4 | 4-6h/d | 1.75(0.59, 5.32) | 0.353 |
| Q4 | 6-8h/d | 1.45(0.59, 3.50) | 0.562 |
| Q4 | >8h/d | 0.85(0.33, 2.17) | 0.590 |
| UPF, ultra-processed food; SB, sedentary behavior; OR, odds ratio; 95% CI, 95% confidence interval | | | |

**Supplementary Table 2**. Association Analysis of UPF Intake, SB, and Depressive Symptoms After Categorization for Age and BMI.

| **Variables** | **Model 1** | | **Model 2** | | **Model 3** | |
| --- | --- | --- | --- | --- | --- | --- |
|  | ***OR*(95%*CI*)** | ***P*** | ***OR*(95%*CI*)** | ***P*** | ***OR*(95%*CI*)** | ***P*** |
| **UPF intake** |  |  |  |  |  |  |
| Q1 | 1.00 |  | 1.00 |  | 1.00 |  |
| Q2 | 1.41(1.04-1.92) | 0.029 | 1.34(0.98-1.84) | 0.070 | 1.26(0.91-1.74) | 0.157 |
| Q3 | 1.98(1.46-2.68) | <0.001 | 1.78(1.30-2.43) | <0.001 | 1.59(1.15-2.19) | 0.005 |
| Q4 | 2.62(1.93-3.54) | <0.001 | 2.29(1.67-3.15) | <0.001 | 2.00(1.44-2.77) | <0.001 |
| **SB** |  |  |  |  |  |  |
| <4h/d | 1.00 |  | 1.00 |  | 1.00 |  |
| 4-6h/d | 0.88(0.62-1.25) | 0.480 | 0.97(0.67-1.39) | 0.850 | 0.94(0.65-1.37) | 0.747 |
| 6-8h/d | 0.99(0.74-1.33) | 0.964 | 1.07(0.79-1.46) | 0.654 | 1.09(0.80-1.49) | 0.600 |
| >8h/d | 1.61(1.19-2.18) | 0.002 | 1.80(1.30-2.48) | <0.001 | 1.75(1.25-2.44) | 0.001 |
| **Note**: Logistic regression models were used to calculate odds ratios (ORs) and 95% confidence intervals (CIs).  ·Model 1: unadjusted.  ·Model 2: adjusted for age (≤25/ >25), gender, education level, income status, history of mental illness, medication use for mental health conditions and BMI (<18.5/ 18.5-24/ ≥24).  ·Model 3: further adjusted for lifestyle behaviors (smoking, alcohol consumption, sleep duration, physical activity) and total energy intake.  Abbreviations: UPF, ultra-processed food; SB, sedentary behavior. | | | | | | |

**Supplementary Table 3**. Analysis of the combined effect of UPF intake and SB on depressive symptoms after categorization for age and BMI.

| **Group** | **n(%)** | **Model 1** | | **Model 2** | | **Model 3** | |
| --- | --- | --- | --- | --- | --- | --- | --- |
|  |  | ***OR*(95%*CI*)** | ***P*** | ***OR*(95%*CI*)** | ***P*** | ***OR*(95%*CI*)** | ***P*** |
| A | 242(16.6) | 1.00 |  | 1.00 |  | 1.00 |  |
| B | 488(33.4) | 1.50(1.07-2.09) | 0.019 | 1.52(1.08-2.15) | 0.018 | 1.47(1.03-2.09) | 0.033 |
| C | 291(19.9) | 2.18(1.52-3.14) | <0.001 | 1.94(1.34-2.81) | <0.001 | 1.69(1.15-2.48) | 0.007 |
| D | 440(30.1) | 2.75(1.97-3.86) | <0.001 | 2.56(1.81-3.63) | <0.001 | 2.29(1.60-3.28) | <0.001 |
| **Note**: Logistic regression models were used to calculate odds ratios (ORs) and 95% confidence intervals (CIs).  ·Group A: Low UPF intake and sedentary time <6 hours/day (Reference group)  ·Group B: Low UPF intake and sedentary time ≥6 hours/day  ·Group C: High UPF intake and sedentary time <6 hours/day  ·Group D: High UPF intake and sedentary time ≥6 hours/day  Model adjustments:  ·Model 1: unadjusted.  ·Model 2: adjusted for age (≤25/ >25), gender, education level, income status, history of mental illness, medication use for mental health conditions and BMI (<18.5/ 18.5-24/ ≥24).  ·Model 3: further adjusted for lifestyle behaviors (smoking, alcohol consumption, sleep duration, physical activity) and total energy intake.  Abbreviations: UPF, ultra-processed food; SB, sedentary behavior. | | | | | | | |
